# *Drosophila melanogaster* in the Study of Human Neurodegeneration

**DOI:** 10.2174/187152710791556104

**Published:** 2010-08

**Authors:** Frank Hirth

**Affiliations:** King's College London, MRC Centre for Neurodegeneration Research, Institute of Psychiatry, Department of Neuroscience, PO Box 37, 16 De Crespigny Park, London SE5 8AF, UK

**Keywords:** Alzheimer’s disease, Parkinson’s disease, motor neuron disease, trinucleotide repeat expansion disease, c-Jun N-terminal kinase, bone morphogenetic protein, neurodegeneration, *Drosophila*.

## Abstract

Human neurodegenerative diseases are devastating illnesses that predominantly affect elderly people. The majority of the diseases are associated with pathogenic oligomers from misfolded proteins, eventually causing the formation of aggregates and the progressive loss of neurons in the brain and nervous system. Several of these proteinopathies are sporadic and the cause of pathogenesis remains elusive. Heritable forms are associated with genetic defects, suggesting that the affected protein is causally related to disease formation and/or progression. The limitations of human genetics, however, make it necessary to use model systems to analyse affected genes and pathways in more detail. During the last two decades, research using the genetically amenable fruitfly has established *Drosophila melanogaster* as a valuable model system in the study of human neurodegeneration. These studies offer reliable models for Alzheimer’s, Parkinson’s, and motor neuron diseases, as well as models for trinucleotide repeat expansion diseases, including ataxias and Huntington’s disease. As a result of these studies, several signalling pathways including phosphatidylinositol 3-kinase (PI3K)/Akt and target of rapamycin (TOR), c-Jun N-terminal kinase (JNK) and bone morphogenetic protein (BMP) signalling, have been shown to be deregulated in models of proteinopathies, suggesting that two or more initiating events may trigger disease formation in an age-related manner. Moreover, these studies also demonstrate that the fruitfly can be used to screen chemical compounds for their potential to prevent or ameliorate the disease, which in turn can directly guide clinical research and the development of novel therapeutic strategies for the treatment of human neurodegenerative diseases.

## INTRODUCTION

Human neurodegenerative diseases describe a clinical condition characterised by the selective and progressive loss of neurons, eventually leading to cognitive, behavioural and physical defects that can cause the death of the patient [1]. Neurodegenerative diseases are categorised by clinical appearance and pathology, such as movement disorders (e.g. Parkinson’s (PD), Huntington’s (HD), motor neuron diseases (MND)) and dementias (e.g. Alzheimer’s disease (AD), fronto-temporal dementia (FTD)); by the type(s) of neurons affected, such as dopaminergic (PD), GABAergic (HD) or motor neurons (MND); and by means of origin as to whether the form of disease is heritable (familial cases) or not (sporadic cases). Age is the greatest risk factor and adult-onset neurodegenerative diseases present a growing socio-economic burden for developed societies because of increased life expectancy. Current estimates for the number of individuals suffering, for example, from AD are as high as 16 million across the United States, France, Germany, Italy, Spain, United Kingdom, and Japan, and the prevalence of AD is estimated to exceed 21 million patients by 2010 [2].

For the vast majority of neurodegenerative diseases, the causes are unclear and effective treatments are lacking. At its best, prescribed treatments such as acetylcholinesterase inhibitors (AD treatment) [3] or levodopa (PD treatment) [4] provide modest symptomatic relief in a proportion of patients. To date, no drug has been identified that does more than improve symptoms. This is particularly exigent for those 95% of patients that suffer from a sporadic form of disease. In those sporadic cases, no indication allows a decided inference about the underlying causes as well as the pathogenic mechanisms involved, apart from age as the main risk factor. This lack of mechanistic insights has been challenged over the last two decades by two different but mutually overlapping approaches.

The first approach addresses the nature and content of proteinaceous inclusions that are typical pathological features of the majority of neurodegenerative diseases, including AD, PD, FTD, several trinucleotide repeat expansion diseases (TRED) and MND. These inclusions are characterised by protein aggregates that accumulate in the extracellular milieu or intracellular compartments of affected neurons. The proteins are usually modified (fragmented, phosphorylated, ubiquitinated) and their identification provides a mechanistic link to the potential aberrant pathway mediating pathogenesis. Textbook examples are amyloid plaques in AD that comprise fragments of aberrantly cleaved amyloid precursor protein (APP) [5-8], Lewy bodies in PD that comprise forms of alpha-synuclein (ASYN) [9], tangles in AD, FTD, PD and MND that comprise forms of tau [10-14], and more recently TAR DNA binding protein 43 (TDP-43) containing aggregates in MND, FTD, AD and PD [15, 16]. It is a striking mystery, however, that comparable inclusions can be observed in several diseases, such as tangles in AD and MND, although present in different neuronal cell types. This enigma as well as clinical evidence suggesting that neurodegeneration can occur even in the absence of inclusion formation continues to fuel the debate as to whether aggregate formation is a cause of disease or rather an attempt of the cell to protect itself. In any case, the identification of the nature and content of proteinaceous inclusions alone has not revealed a major breakthrough in understanding disease formation. However, in conjunction with information generated by the second approach, these data led to the identification of key pathogenic pathways operant in human neurodegeneration.

The second approach addresses familial forms of neurodegene-rative diseases, even though they represent only the minority of about 5% of all cases. Familial cases show clinical features similar to sporadic cases but at the same time are heritable, substantiating the reasonable quest to identify the origin, cause and underlying mechanisms of disease. Accordingly, large-scale pedigree analyses and genome-wide association studies have been and are still carried out in order to identify genes and loci that are affected in neurodegenerative diseases [see recent example,  17,  18]. These efforts led to the identification of APP, tau, ASYN, Huntingtin (HTT), and TDP-43 as the major culprits involved in AD, FTD, PD, HD, and MND, respectively. The genetic data corroborate earlier observations that proteinaceous inclusions harbour Abeta fragments derived from aberrant APP cleavage (amyloid plaques), tau (tangles), ASYN (Lewy bodies), polyglutamine expanded HTT and TDP-43. The fact that inclusion content and a mutant allele converge on the same defective protein unifies familial and sporadic cases and strongly suggests that the affected protein is causally related to disease formation and/or progression.

The limitations of human genetic studies however, often make it difficult to analyse genes and pathways in any further detail, because of complex patterns of inheritance, lack of sufficient family pedigree data and population-based genetic heterogeneity. Therefore, model systems are used to study specific functional aspects of the genes/proteins identified in neurodegenerative diseases. These models range from yeast [19] and *C. elegans* [20] to mammals and human cell culture systems. But yet, in most of the cases, these models fulfil only some of the criteria expected to apply in the study of human neurodegeneration. Essential criteria include: cognitive, behavioural and physical dysfunction caused by cell type-specific neurodegeneration; cellular pathophysiology including aggregate formation; clear pattern of inheritance; pedigree data covering three and more generations; population-based genetic homogeneity. In addition, an ideal model system would provide further detailed information: case-specific data spanning a whole life from conception to age-related death, which would allow the study of disease formation and progression in relation to age as the major risk factor; knowledge about the focus of disease, its time and site of origin, which in turn would allow the visualisation and eventually manipulation of disease formation and progression; and large numbers of genetically identical individuals, like multiple twins, that would allow strong power calculations to deduce quantitative traits as well as insights into pathogenic mechanisms in a statistically significant manner. An organism that meets all these criteria in a formidable way is the fruitfly *Drosophila melanogaster*.

## DROSOPHILA MELANOGASTER

The protostomian, ecdysozoan arthropod *Drosophila melanogaster* belongs to a sub-species of the *Drosophilidae*, dipteran insects that are found all over the globe. During the course of evolution, the arthropod lineage already separated from the vertebrate lineage more than 600 million years ago [21, 22], suggesting that *Drosophila* might be completely unrelated to humans. However, genetic, molecular and behavioural analyses over more than a century suggest otherwise, and *Drosophila* has been used as a prime model organism for experimental studies of multi-cellular eukaryotic biology. This led to the discovery of fundamental biological principles, such as validation of the chromosomal theory of inheritance and the first experimental description of the gene as a functional unit [23].

**Fig. (1):**
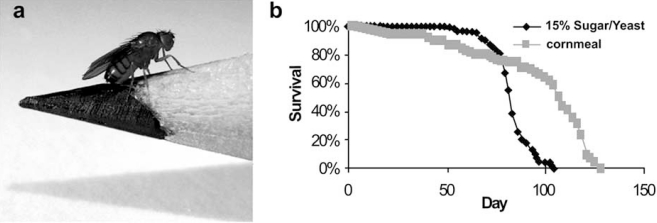
***Drosophila* as a model organism in the study of age-related neurodegeneration**. (**a**) The arthropod Drosophila melanogaster belongs to a subspecies of the *Drosophilidae*, dipteran insects that fit on a pencil tip and can be easily kept *en masse* in the laboratory. Their anatomy displays characteristic features such as compound eyes, wings and bristles that can be used as phenotypes to study neurodegeneration without affecting the survival of the fly. (**b**) The lifespan of *Drosophila* depends on diet and stress and varies between 40-120 days. The more rigid a diet (e.g. cornmeal), the longer a fly can live, whereas increase in carbohydrates and cholesterol (e.g. 15% sugar/yeast) can lower life expectancy. These similarities to human ageing and lifespan, together with a highly conserved genetic makeup, make *Drosophila* a powerful model system in the study of adult-onset, age-related neurodegeneration.

**Fig. (2):**
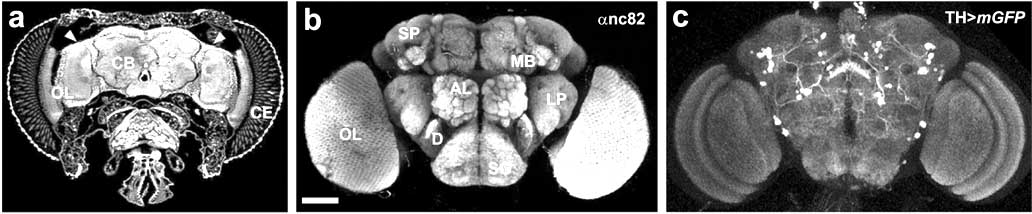
**Adult brain of *Drosophila***. (**a**) Confocal image of a parafin cross-section through the adult *Drosophila* head; auto-immunofluorescence visualises the ommatidia of the compound eye (CE), the optic lobe (OL) and the central brain (CB). Note that cell bodies (arrowheads) are topologically separated from axonal extensions which make up the neuropil. (**b**) Confocal image of a whole mount adult brain immunolabelled with anti-nc82 which recognises the Bruchpilot protein that is specifically enriched in active zones of synaptic terminals. This allows the visualisation of cortical areas in the fly brain, including optic lobes (OL), antennal lobes (AL), superior protocerebrum (SP), lateral protocerebrum (LP), mushroom bodies (MB), deuterocerebrum (D), and subesophageal ganglion (SG). (c) Optical cross-section of a whole-mount adult brain of a transgenic Drosophila immunolabeled with anti-nc82; tyrosine hydroxylase (TH)-specific Gal4 drives *UAS-mCD8:GFP* expression, a membrane-tagged GFP (*TH*>*mGFP*). Because TH is the rate-limiting enzyme of dopamine synthesis, this transgenic Gal4/UAS combination visualises dopaminergic neurons and their axonal extensions (white/light grey). Based on this method, dopaminergic neurons can not only be monitored, but also manipulated, and cell numbers as well as axonal projections can be used as phenotypic read-out parameters to study parkinsonism in *Drosophila*. Scale bar: 50 µm.

Apart from tradition, the reasons for using the fruitfly as a study object are manifold: *Drosophila* is cheap and easy to maintain in the laboratory (Fig. **1a**); it can give rise to a large number of genetically identical progeny; it has a rather short life span ranging from 40 to 120 days (Fig. **1b**) depending on diet and stress [24, 25]; it shows complex behaviour, including learning and memory [26, 27], driven by a sophisticated brain (Fig. **2**) and nervous system [28]. The entire *Drosophila* genome is encoded by roughly 13,600 genes as compared to 27,000 human genes, located on only four pairs of chromosomes as compared to 23 pairs in human [29]. Thanks to very well-described anatomy and development [30, 31], and the availability of molecular genetic tools [32-34], *Drosophila* is one of the most extensively used genetic model organisms to study complex biological processes. In comparison to other organisms like *C. elegans* and the mouse, the fly provides a very powerful genetic model system for the analysis of brain and behavioural disorders related to human disease: its brain is complex enough (as compared to *C. elegans*) to make fly behaviour highly interesting and relevant to humans but it is still small enough (as compared to mouse) for an in-depth structural and functional analysis [35].

The value of *Drosophila* as a model system has been amply demonstrated by the fact that many genes and processes first discovered in the fly have proven to be conserved in other organisms, including humans. Comparative analysis of whole genome sequencing revealed striking similarities in the structural composition of individual genes of* Homo sapiens* and *Drosophila* [36]. Moreover, the molecules and mechanisms underlying core modules of cell biology are conserved as well: homologous genes mediate homologous pathways such as cyclin/cdk modules regulating the eukaryotic cell cycle [37-39], or insulin signalling regulating metazoan cell growth [40, 41]. These data provide compelling evidence for the structural conservation of genes due to common origin; they elucidate a deep homology underlying cell biological mechanisms that extends beyond gene structure to patterned protein expression and function. This notion is further supported by experiments demonstrating that *Drosophila* and human genes can substitute each other in species-specific but evolutionarily conserved mechanisms underlying brain development in insects and mammals [42-45].

Based on these observations, it is obvious that *Drosophila* can offer unique opportunities in the study of human neurodegeneration: Most of the genes implicated in familial forms of disease have at least one fly homolog [46] (see Tables **1-4**); fundamental cellular processes related to neurobiology are similar in *Drosophila* and humans, including synapse formation, neuronal communication, membrane trafficking and cell death; the neurobiological bases of behaviour are of the same kind in flies and humans, including sensory perception, integration and motor output, as well as aspects of learning and memory formation. These features make *Drosophila* a prime model organism in the study of adult-onset, age-related neurodegeneration.

## *DROSOPHILA* IN THE STUDY OF NEURODEGENERATION

The fundamental aims in the study of neurodegeneration are to elucidate underlying pathogenic pathway(s) and in turn the development and successful application of targeted treatment(s) to stop or at least ameliorate the disease. The genetic dissection of disease pathways is therefore a reasonable means to address these aims and three mutually interrelated approaches are utilised in *Drosophila*:

- mis-expression of a human disease gene, in its wild-type or mutant form;
- loss and gain of function of the *Drosophila* homolog of a human disease gene;
- genetic screens to identify enhancers and suppressors that are able to modify a phenotype caused by (i) and (ii).

Mis-expression of a human gene is used to investigate its functional properties in a neurodegenerative process and to elucidate its interactions with other *Drosophila* proteins. This is usually achieved by using the Gal4/ upstream activating sequence (UAS) system that allows time- and tissue-specific mis-expression of any gene of interest in *Drosophila* [47]. Most robust models have been established using nervous system-specific Gal4 drivers, thereby corresponding to the human condition. However, several other tissues are also used as a phenotypic read-out system, including the compound eyes, wings and bristles, because a degenerative phenotype can be revealed without affecting the survival of the fly. Especially the compound eye is predominantly used because it allows the generation of a neurodegenerative phenotype (rough eye phenotype) that can be easily scored under a standard light microscope [see, for example,  48], and in turn can be utilised for genetic enhancer/suppressor screens. 

There are, however, serious drawbacks to this approach: (i) degeneration can occur by excess Gal4 protein itself [49], thereby causing a rough eye phenotype unrelated to the disease gene under investigation; (ii) a rough eye phenotype can occur by degeneration of accessory cells unrelated to *neuro*degeneration; (iii) the severity of a degenerative phenotype may not relate to the amount of protein expressed because of the way the UAS construct (harbouring the human disease gene) has been integrated into the fly genome (*position effect*). The latter can and should be avoided by site-specific genomic integration of the UAS construct (for details, see [50]) so that the same levels of protein expression are achieved. In comparison to wild-type protein expression, only this will permit meaningful conclusions about the impact of mis-expressing a human mutant protein or its post-translational modifications that frequently occur in neurodegeneration, such as hyperphosphorylation or ubiquitination.

The second approach applies standard analytical techniques available to study the function of a *Drosophila* gene/protein. These include mutational inactivation for a loss-of-function analysis, and Gal4/UAS-mediated RNA interference for a knockdown or over-expression for gain-of-function analyses, with several modifications and more sophisticated possibilities available [28, 51-55]. The ultimate goal is to gain insights into the role and function of a *Drosophila* homologue of a human disease gene. By way of homology between the fly and human protein, a reasonable inference can be made on the function of the human protein, and hypotheses and predictions can be deduced about the potential pathogenic pathway(s). Following this approach, it has been shown first in *Drosophila* that mitochondrial dysfunction can result from defective PINK1/Parkin signalling which appears to be one of the pathogenic pathways underlying PD [56-58].

A third approach is utilised once a neurodegenerative phenotype is established.* Drosophila* offers the advantage of performing unbiased genetic screens to identify enhancers and suppressors on a genome-wide basis. Several labs have used the ease and accessibility of the compound eye to carry out such modifier screens, and targeted genes that either suppress or enhance the rough eye phenotype, thereby signifiyng the candidate genes as members of a common pathogenic pathway. These attempts have identified several ‘key’ interacting partners of tau, Abeta and ASYN [59-65]. However, in only very few cases [62, 66], the results obtained in *Drosophila* led to the identification of a human homologue that is similarly involved in the corresponding human disease. The potential reason(s) for such a meagre yield are phenotypic screens predominantly based on eye-specific Gal4 drivers that are already active during development, thereby generating a rough eye phenotype in the newly hatched adult fly - a situation that does not mimic nor model adult-onset neurodegeneration in an age-related manner.

**Fig. (3):**
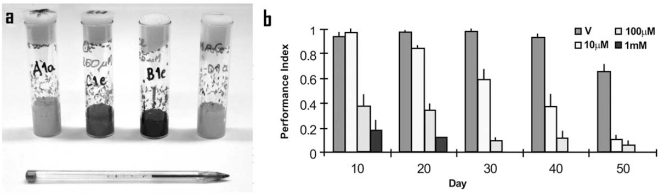
**Drug treatment in *Drosophila***. (**a**) Four vials with flies are kept on cornmeal food each of which has been supplemented with a different concentration of the same drug. The applied drug is screened for its potential to either enhance or suppress a given neurodegeneration phenotype that has been caused by targeted genetic manipulation, such as rough eyes caused by mis-expression of human tau, a movement disorder caused by dysfunction of *Drosophila TDP-43*, or reduced lifespan caused by mis-expression of human ASYN. In this way, *Drosophila* models of neurodegeneration can be used to screen compound collections for their potential to prevent or ameliorate a specific neurodegenerative “disease”. (**b**) Three different concentrations of a drug (10 µM, 100 µM, and 1 mM) are chronically applied to ageing *Drosophila*, as compared to vehicle treated flies. The resulting effects on locomotion are quantified using a negative geotaxis assay: flies are shaken to the bottom of a vial/cylinder; their innate behaviour triggers them to move upwards (against geotaxis) and the time it takes them to reach the top is scored for a cohort of flies and multiple replicates. A calculus then determines the relative performance of these flies exposed to a given drug concentration and at a specific day. The graph shows that the geotaxis performance inversely correlates with drug concentration and age, suggesting that this drug enhances a movement disorder in a concentration-dependant and age-related manner.

In addition to these genetic approaches, a fourth one uses established fly models of neurodegeneration in order to screen compound collections for their potential to prevent or ameliorate the “disease”. A *Drosophila* compound screen is achieved by simply feeding flies with their usual food to which a defined concentration of the compound has been added (Fig. **3**). There are obvious drawbacks to such screens, especially when used for adult onset, age-related neurodegeneration phenotypes, as these screens are time-consuming and far from being “high-throughput”. In addition, compound screens have been ineffective when based on a rough eye phenotype that is generated during development and detectable in the newly hatched adult fly [Luz and Hirth, unpublished]. This is because flies can be easily raised on drug-treated food but ingestion stops during puparium formation and the subsequent pupal stage, which lasts four days until the adult fly hatches. Moreover, this final stage of development is characterised by a high metabolic rate related to metamorphosis, during which a previously incorporated drug loses its efficacy.

Despite all these limitations, *Drosophila* has been used successfully to identify compounds that not only improve symptoms, but also modify the course of the “disease”. These successful cases are mainly (but not only) based on fly models of adult-onset, age-related neurodegeneration, and resulted in improvements relevant to human disease, including extended life-span in models of AD [67] and prolonged survival of dopaminergic neurons in models of PD [68, 69] as well as the complete rescue of disease-related phenotypes [70]. The relevance and impact of such *small-scale* compound screens in *Drosophila* cannot be rated highly enough because of their potential for translational research: the fruitfly is a complex behaving animal with a sophisticated, centralised nervous system including a blood-brain barrier [71], and therefore superior to cell cultures and *C. elegans*, but still inexpensive and short-lived as compared to mice. Accordingly, *Drosophila* drug screens in cancer research [72] have already identified compounds that are now in phase I and II clinical trials, illustrating the enormous potential for its application in the study of human diseases.

The above mentioned experimental approaches have established *Drosophila* as an excellent model organism in the study of human neurodegeneration. Numerous reviews have covered detailed aspects of this topic and are highly recommended to the reader [73-85]. In the following article, key and novel findings as well as their implications are summarized and reviewed. A special emphasis is given to *Drosophila* models of AD, PD, TREDs and MNDs.

## ALZHEIMER’S DISEASE

Alzheimer’s disease is the most common neurodegenerative disease affecting primarily elderly people. Over 95% of the known cases are sporadic, whereas less than 5% account for familial cases [86, 87]. These heritable cases led to the identification of genes that, when mutated appear to be involved in AD pathogenesis, including APP, presenilins 1 and 2 (PSN-1/2) and tau (Table **1**). In addition, Apolipoprotein E isoform epsilon4 has been identified as a risk factor [88]. AD is characterized by progressive memory loss and the subsequent degeneration of large areas of the brain [89, 90]. Microscopically, AD pathology reveals neuritic plaques, composed mainly of Abeta peptides, and neurofibrillary tangles, composed of abnormal tau protein [86, 91, 92]. Abeta peptides are produced by proteolytic cleavage of the APP transmembrane receptor at the beta and gamma sites. Familial mutations (familial AD, FAD) in APP result in increased production of Abeta42 peptide, the amyloidogenic form of the two Abeta species, Abeta40 and Abeta42. Abeta42 forms protofibrils and fibrils much more readily than Abeta40 and is the predominant form of the peptide found in plaques. The membrane-tethered aspartyl protease beta-site APP-cleaving enzyme (BACE) cleaves APP at the beta site, and the presenilins, PS1 and PS2, participate in APP cleavage at the gamma site along with the genes *nicastrin*, *Aph-1* and *Pen-2*. These data led to the amyloid cascade hypothesis as the main culprit of AD formation [93, 94]. *Drosophila* carries homologues of AD-related genes, including APP, presenilin, and tau (Table **1**), which has made it a model system in AD research.

## APP AND ABETA

The *Drosophila* homologue of APP, *β-amyloid protein precursor-like* (*Appl*, CG7727), is expressed in the central nervous system; however, mutational inactivation of *Appl* does not cause a neurodegenerative phenotype [95]. Flies deleted for the *Appl* gene are viable, fertile, and morphologically normal, yet they exhibit subtle behavioural deficits: a fast phototaxis defect can be observed which is partially rescued by wild-type, but not mutant APPL. There is functional homology between APPL and human APP, as transgenes expressing human APP show a similar level of rescue as the fly protein. *Drosophila* APPL lacks homology to APP within the Abeta peptide region, and thus is not cleaved like mammalian APP, and Abeta deposition does not occur *in Drosophila*. Interestingly, however, a recent report identified a beta-secretase-like cleavage site in APPL [96], but further proof is required to show that this is a functional site leading to Abeta-like deposition. Accordingly, *Drosophila* models of APP-mediated AD have used the GAL4/UAS-system to over-express human forms of Abeta.

These models recapitulate, at least to some extent, aspects of human AD pathology, including Abeta plaque deposition [97, 98], defective axonal transport [99, 100] and axonopathies [101], mitochondrial mislocalisation [102], defects in synaptic plasticity [103] and progressive locomotor deficits [98], affected life-span [97, 98, 104], and age-dependent neurodegeneration including vacuolization of the brain [97, 98, 105]. The severity of these phenotypes is related to Abeta toxicity, with the Abeta 42 arctic mutation being the most toxic form.

*Drosophila* Abeta models were subsequently utilised to target modifiers of AD pathology by genetic or pharmacological interference. Genetic experiments showed that secretase inhibitors and neprilysin can ameliorate Abeta42 phenotypes, by either reducing Abeta production [67, 104, 106] or by increasing Abeta degradation [107, 108]. In addition, a recent study identified FKBP52, a prolyl-isomerase of the immunophilin/FK506 binding protein family as a modifier of Abeta toxicity. This study also showed that mutations in the copper transporter Atox1 which interacts with FKBP52 enhance Abeta pathology which can be suppressed in FKBP52 mutant flies raised on a copper chelator diet [109]. Pharmacological interference identified Congo Red [104] and glutaminyl cyclase inhibitors [110] as effective suppressors of Abeta deposition and amyloid plaque formation.

Resulting from these studies, several signalling pathways have been identified as potential mediators of Abeta pathogenesis. These include altered Toll->NFkappaB signalling [65], age-dependent autophagic-lysosomal injury [111], and the Abelson tyrosine kinase/JNK stress kinase cascades [112]. Autophagy and JNK signalling have been shown to be altered in human AD [113, 114]. However, it is not clear whether these pathways are activated because of a causal relationship to disease formation/progression or because of a cellular protection attempt. In any case, these studies not only corroborate the amyloid cascade hypothesis, but also support recent data indicating that Abeta oligomerization, rather than plaque formation, is the toxic event that acts as a seed for Abeta aggregation [110, 115, 116]. Yet, it is unknown how Abeta oligomerization causes neuronal cell death.

## PRESENILIN AND FAD

FAD mutations in *PS1* and *PS2* alter proteolytic processing of APP to generate more toxic Abeta42 peptides which accelerates amyloid plaque formation in brain tissues. PS mutations also contribute to neurodegeneration and cognitive decline through amyloid-independent mechanisms, involving altered regulation of receptor signalling and intracellular kinase activity. *Drosophila Presenilin* (*Psn*, CG18803) represents the only fly homologue of the mammalian PSN1 and PSN2 genes. Functional studies in *Drosophila* showed that it is involved in various biological processes: cytoskeleton organization and biogenesis; cell fate commitment; nervous system development; Notch receptor processing; membrane protein ectodomain proteolysis; cell-cell adhesion and intracellular signalling cascades. With regard to neurodegeneration, *Drosophila Psn* is not involved in Abeta42 production because APPL lacks homology to APP within the Abeta peptide region, and thus is not cleaved like mammalian APP. Accordingly, a more suitable substrate to monitor Psn FAD mutant activity in *Drosophila* is the Notch receptor, the most extensively characterized fly gamma-secretase substrate [117]. *Psn* is required throughout *Drosophila* development for Notch signalling, and a wide variety of Notch-related phenotypes exist that range from severe embryonic lethality to specific defects in adult tissues [118].

As is the case for Abeta models, Psn1/2 wild-type and mutant forms have been mis-expressed in *Drosophila*. These studies reveal that PSN FAD mutant activities are tightly linked to the age-of-onset of degeneration, suggesting that disease severity primarily reflects differences in PSN mutant lesions [119]. Most recent studies relate PSN-mediated pathogenesis to* ubiquilin* dysfunction [120, 121] and defective calcium storage that can be suppressed by *calmodulin* loss-of-function mutations [122]. The latter model underscores earlier findings suggesting that perturbed neuronal Ca^2+^ homeostasis is implicated in PSN and APP-mediated AD pathogenesis, whereby the resulting toxic forms of Abeta can induce Ca^2+^ influx into neurons. This occurs by Abeta forming an oligomeric pore in the membrane which in turn renders neurons vulnerable to excitotoxicity and apoptosis (for review see [123]). However, as is the case for Abeta models, the actual cause(s) of cell death are currently unknown, although defects in axonal transport and synaptic dysfunction refer to cytoskeletal abnormalities that are the major culprit of AD-related tauopathy.

## TAU AND TAUOPATHY

Pathological aggregation of the microtubule-associated protein tau [124] is a defining feature not only of AD but also of other neurodegenerative diseases collectively called tauopathies [91, 92, 125]. The process of tau accumulation, paired helical filament assembly, and aggregation is incompletely understood. While tau hyper-phosphorylation clearly accelerates neurodegeneration, the role of other posttranslational modifications, including proteolysis, ubiquitination, nitration, and glycosylation, as well as the function of the tau amino terminus in this process, remain unclear.

The *Drosophila* homologue of MAPtau,* tau* (CG31057) exhibits 46% identity and 66% similarity with the human protein. However, the fly protein does not contain the N-terminal repeats found in several human isoforms of *tau* [126]. To date, *Drosophila* models of tauopathy rely on the over-expression of wild-type or mutant forms of human tau, including the FTD-related P301L, V337M and R406W mutants. The resulting phenotypes mimic, at least to some extent, the human disease condition, including learning and memory deficits [127], phosphorylation–dependent tau toxicity [128, 129], correlation between FTD-related mutant forms and enhanced toxicity [130, 131], tangle formation in some [132] but not all cases [see, f.e.  130], synergistic interaction with Abeta leading to Hirano body formation [131], and altered life-span as well as region-specific neurodegeneration in the adult brain [130-132].

Subsequent genetic interaction studies showed that kinase-dependent phosphorylation increases tau toxicity and identified Shaggy/glycogen synthase kinase-3 [132] and MARK/Par-1 [129, 133] as key players in this process. In addition, genetic modifier screens identified targets and signalling pathways as potential mediators of tau toxicity, including puromycin-sensitive aminopeptidase [134], altered Wingless [132] and JNK [135] signalling, as well as TOR-mediated cell cycle activation [136]. Although it is still unclear whether aberrant cell cycle activation is directly causing tauopathy/neurodegeneration (see, for example, [137]), a potential role of defective TOR signalling has been further substantiated by pharmacological interference. These data show that rapamycin targeting TOR is able to reduce wild-type or mutant tau-induced toxicity by reducing insoluble tau [138]. Yet, the relevance of puromycin-sensitive aminopeptidase and TOR signalling in human AD pathogenesis remains to be established. In contrast, pathological tau phosphorylation has been shown to be involved in human disease progression [139], and there is at least circumstantial evidence that altered JNK signalling may contribute to AD formation and/or progression [114].

## PARKINSON’S DISEASE

Parkinson’s disease is the most common neurodegenerative movement disorder characterized by severe motor symptoms, including uncontrollable tremor, imbalance, slowness of movement and rigidity. Among the exemplary pathological changes observed in PD is the progressive loss of dopamine (DA) neurons in the substantia nigra pars compacta of the ventral midbrain, although neuropathology is not limited to this region [89]. DA neurons of the substantia nigra pars compacta innervate the putamen and caudate via the nigrostriatal pathway and thereby exert a stimulating function to the striatum regulating motor control. Loss of DA neurons and the subsequent degeneration of the nigrostriatal pathway are a primary cause for movement disorders observed in PD cases. DA cell loss is usually associated with the presence of intraneuronal inclusions known as Lewy bodies, which are composed principally of ASYN [9].

PD prevalence increases with age, with a mean age of onset around 70 years, although 4% of patients develop early-onset disease before the age of 50 [140]. The mean disease duration from diagnosis to death is 15 years, but the precise mode of death is often difficult to determine. Males appear to be 1.5 times more likely to develop PD than females, although the underlying causes are not known [141]. PD is progressive and current treatment is symptomatic only with DA (levodopa) replacement as the major therapy. The majority of PD cases are sporadic, likely to be caused by a combination of risk factors, the most evident being age. However, there are also rare familial disease forms caused by gene mutations which show similar clinical and neuropathological features. Although these inherited forms account for 5% of all PD cases only, studies of the function of the affected genes have provided insights into PD pathogenesis.

Several genetic loci have been identified that are affected in familial forms of PD or have been identified as being associated with PD. These include (see Table **2**): *alpha-synuclein (asyn)*, *parkin*, *ubiquitin carboxy-terminal hydrolase L1* (*UCHL1)*, *phosphatase and tensin homologue (PTEN)-induced kinase 1,* (*PINK1),* *DJ-1*, *leucine-rich-repeat kinase 2* (*LRRK2)*, *high temperature requirement protein A2* (*HTRA2), glucocerebrosidase, polymerase gamma *and* tau* [141-146]. Based on pathophysiology as well as genetic defects, three types of cellular dysfunction are currently implicated in the pathogenesis of PD: abnormal protein aggregation, oxidative damage, and mitochondrial dysfunction [142, 143, 145, 147, 148]. Homologues for PD genes exist in *Drosophila*, with the interesting exception of ASYN (see Table **2**). Accordingly, *Drosophila* models of PD have been established based on the experimental approaches outlined above. Rather than listing them gene by gene, I consider it more reasonable to summarise the prerequisites, main findings and resulting insights into PD pathogenesis.

## PARKINSONISM IN *DROSOPHILA*

Synthesis of the neurotransmitter DA is conserved between *Drosophila* and human and distinct clusters of DA neurons are detectable in the developing and adult fly brain [149]. Comparable to the human condition, the *Drosophila* DA system is also involved in locomotor control [150, 151], although the details of the underlying neural circuit(s) are unknown. It is therefore reasonable to assume that loss of DA neurons can affect locomotion in *Drosophila *comparable to the situation in PD*.* Indeed, loss of subsets of DA neurons in the brain as well as locomotion defects are the two principal parkinsonian-like phenotypes used to characterise fly models of PD. Both phenotypes have been induced by mis-expression of wild-type and/or mutant forms of human PD genes, including ASYN [152-160], PINK1 [159, 161, 162], Parkin [154-156, 163, 164] and LRRK2 [165-169].

Loss-of-function mutations or DA neuron-specific inactivation of the respective *Drosophila* homologues of PINK1 [161, 170-173], Parkin [61, 174-176], DJ-1 [69, 177-182], LRRK2 [165, 166, 173, 183], and HtrA2 [184-186] can also lead to parkinsonism-like DA cell loss and locomotor defects. However, it should be noted that in several cases loss of the *Drosophila* homologue of a human PD gene causes a much weaker phenotype than mis-expression of the corresponding human protein. Moreover, for some models including LRRK2 and HtrA2 there are conflicting data as to whether loss of the *Drosophila* homologue causes a parkinsonian-like phenotype or not [166, 183, 185, 186]. These discrepancies might be attributable, at least to some extent, to the inherent artificial situation of Gal4-mediated protein overload added to the endogenous protein level. In addition, the total number of DA neurons per cluster varies between individual wild-type flies [White and Hirth, unpublished] and the methods used to measure DA cell loss can substantially impact on the phenotypic read-out [187].

In addition to these genetic modifications, pharmacological insults also cause parkinsonian-like phenotypes in *Drosophila*, thereby modelling sporadic cases of PD. The pesticide rotenone as well as paraquat-induced oxidative damage cause cluster-specific DA neuron loss together with motor deficits that can be ameliorated by levodopa added to the food [188, 189]. In contrast, a *Drosophila* model of 1-methyl-4-phenyl-1,2,3,6-tetrahydropyridine-induced parkinsonism [for review see  142] has not been established so far.

It should be noted, however, that in *Drosophila *the majority of these genetic and pharmacological models do not reveal Lewy body formation, which is a predominant pathological feature of both familial and sporadic cases of PD. As noted above, a *Drosophila* homologue of ASYN is missing which may account for the lack of Lewy body formation, except for those cases where human ASYN is mis-expressed in the fly [152, 153, 157, 160, 190]. Nevertheless, *Drosophila* models of PD have revealed valuable insights into potential pathogenic mechanisms and identified targets of ASYN and other PD-related genes.

## MECHANISMS AND TARGETS OF PARKINSONISM

ASYN is the principal component of Lewy bodies found in the majority of PD cases and therefore represents a prime target for PD research. Although a role in synapse homeostasis is suggested, its wild-type function remains elusive [191]. Insights into ASYN-mediated pathogenesis come from studies showing that phosphorylation and enhanced oligomer formation are the major culprits of ASYN toxicity in *Drosophila* [157, 190-193]. Thus, phosphorylation at residue serine 129 can ameliorate [190] whereas phospho tyrosine 125 can exacerbate soluble oligomer formation and toxicity [193]. Moreover, a recent elegant study addressing the *in vivo* impact of biophysically defined pre-fibrillar variants of ASYN showed that the increasing potential to form fibrils and soluble oligomers correlates with increasing neurodegeneration, not only in *Drosophila* but also in* C. elegans* and human cell culture models [192]. These data suggest that soluble oligomers are the most toxic species in PD-like pathogenesis, which is further supported by the fact that an ASYN mutant lacking residues 71-82 and unable to form oligomers and fibrils, is also unable to induce DA neurotoxicity in the fly [157].

*Drosophila* ASYN models have been used to target modifiers of PD pathology by genetic or pharmacological interference. Genetic experiments showed that calpain cleavage [194] and superoxide dismutase (SOD) activity [158] affect ASYN, whereas inhibition of Silent information regulator 2 (Sir2), a nicotinamide adenine dinucleotide–dependent histone deacetylase rescues ASYN toxicity [66]. Moreover, ASYN inhibits histone acetylases and its neurotoxic effects can be rescued by histone deacetylase inhibitors [195], further supporting a link between ASYN-mediated pathogenesis and histone acetylation. Pharmacological interference identified nicotinamide which can improve motor deficits [196]; as does dietary supplementation of S-methyl-L-cysteine, a substrate in the catalytic antioxidant system mediated by methionine sulfoxide reductases [197]. In addition, geldanamycin can suppress ASYN toxicity, a process that appears to be mediated by heat shock protein 70 chaperone activity [68].

The latter studies strongly suggest that ASYN-mediated pathogenesis is accompanied, if not accelerated by a stress response, which can be also found in other *Drosophila* models of PD. Loss of PINK1 function in the fly eye can be suppressed by SOD1 as well as with vitamine E [161]. Loss of *parkin* leads to oxidative stress [175] and in both ASYN and parkin models, neuronal cell loss can be overcome by genetic or pharmacological interventions increasing glutathione synthesis or glutathione conjugation activity [198]. This detox activity of parkin might be mediated by glutathione S-transferase S1 [61]. Parkin also associates with parkin-associated endothelin receptor-like receptor, which by itself can cause parkinsonism in *Drosophila* [154, 199]. Similar to ASYN and parkin, DJ-1 has been shown to be involved in an oxidative stress response [69, 177-179, 181, 182]. Some of these screens also indentified signalling pathways that mediate PD-like pathogenesis. Among those involved in Parkin-mediated pathogenesis are PI3K/Akt [180, 200], JNK [176] as well as TOR signalling [167, 173]. Interestingly, both Parkin and LRRK2-mediated pathogenesis can be ameliorated with rapamycin targeting 4E-BP activity [173], a situation comparable to its application in *Drosophila* models of tauopathy [138].

## PD-RELATED MITOCHONDRIAL DYSFUNCTION

A surprising and equally promising finding of these interaction studies in *Drosophila* was the discovery that LRRK2, PINK1 and Parkin genetically interact with each other [168, 173] as does DJ-1 [169]. PINK1 is able to phosphorylate Parkin, which is a requirement for its mitochondrial localisation [201]. PINK1 and Parkin loss-of-function genotypes phenocopy each other as both affect mitochondrial morphology. Subsequent epistatic analyses demonstrated that the mitochondrial phenotype caused by PINK1 loss-of-function can be rescued by Parkin [170-172]. Moreover, over-expression of human Parkin mutant R275W but not wild-type or G328E mutant can cause mitochondrial abnormalities similar to Parkin loss-of-function mutants [163]. These data strongly suggest that at least PINK1 and Parkin act together in a common pathway regulating mitochondrial function [57, 58].

Mitochondria are endosymbiontic organelles found in all eukaryotic cells required for ATP production as well calcium buffering and apoptotic signals [202]; they encode their own DNA (mtDNA) in up to 1000 copies per cell, and undergo frequent morphological changes through fission and fusion, thereby retaining mtDNA integrity and essential neuronal function, such as synaptic transmission, plasticity, and ultimately cell survival [203, 204]. The PD model studies in *Drosophila* showed that PINK1 and Parkin regulate mitochondrial morphology [162, 205, 206], potentially by acting on Drp-1 [207] and other factors mediating mitochondrial fission/fusion. It should be noted, however, that the majority of these interactions have been discovered and described using the fly muscle as a model, and not in DA neurons of the fly brain itself. Moreover, it is currently not known whether PINK1 and/or Parkin act directly or indirectly on genes regulating fission/fusion and mtDNA integrity. PINK1 and Parkin loss-of function increases Drp-1 dependent mitochondrial fragmentation, suggesting that at least a feedback loop exists between PINK1/Parkin signalling and mitochondrial fission/fusion. It remains to be shown whether this feedback loop requires other mediators regulating mtDNA maintenance and/or fission/fusion.

In summary, these examples of *Drosophila* research into human PD corroborate and extend current hypotheses suggesting that abnormal protein aggregation, oxidative damage and mitochondrial dysfunction are causally related to PD pathogenesis. The results obtained in *Drosophila* led to the identification of homologues and homologous pathways involved in the formation and progression of parkinsonism. Thus, despite some heterogeneity, these models identify oligomer formation of ASYN and histone acetylation, as well as altered PINK1/Parkin signalling and mitochondrial dysfunction as two distinguishable pathogenic pathways underlying PD-like neurodegeneration – a situation corresponding to the human disease condition [145]. These data from *Drosophila* also identify JNK as well as PI3K/Akt and TOR signalling as contributors to disease progression, whereas detox pathways reducing oxidative stress, either genetically or pharmacologically, can ameliorate it. Significantly, these pathways parallel some of those involved in AD pathogenesis, indicating that common disease mechanisms may underlie AD and PD-like neurodegeneration.

## TRINUCLEOTIDE REPEAT EXPANSION DISEASES

TREDs account for more than 16 neurological disorders that are caused by aberrant expansion of triplet reiterations in either coding or non-coding regions of disease-specific genetic loci that result in dysfunction of the respective protein, eventually leading to neurodegeneration and, ultimately, patient death (for review see [208]). The majority of TREDs are diseases caused by expansion of CAG repeats coding for glutamine (polyglutamine, PolyQ), including HD, spinal bulbar muscular atrophy (SBMA, also Kennedy disease), spinocerebellar ataxias (SCA) 1, 2, 3 (also known as Machado-Joseph disease), 6, 7, and 17, and dentatorubral-pallidoluysian atrophy (DRPLA). Other TREDs include Fragile X syndrome (FRX, CGGn repeats) and Friedreich’s ataxia (FRDA, GAAn repeats). Apart from codon reiteration as a common denominator, TREDs differ in disease-related length of expanded repeats, age of onset, clinical features and neuropathology. The reader is referred to reviews addressing each TRED in more detail [208-210] The genetic loci affected by the expansion of unstable trinucleotide repeats have been identified and with the exception of the androgen receptor, there is a fly homologue known for each of them (see Table **3**), which in turn led to the establishment of *Drosophila* models of PolyQ diseases, FRX and FRDA.

## POLYQ-RELATED NEURODEGENERATION

HD, SBMA, SCA1/2/3/6/7/17 and DRPLA are predominantly inherited diseases where polyQ expansions confer structural changes to the disease-related protein, leading to oligomeric species and protofibril forms rich in beta-sheets [211]. The resulting mutant proteins become dominantly toxic and can lead to intra-nuclear inclusions in neurons and glial cells, ultimately causing neuronal dysfunction and cellular degeneration. The direct causes of degenerative cell death still remain elusive but there is an inverse correlation between repeat length and disease severity, with expansions of 40 glutamines and more being a general threshold for disease formation [212].

As is the case for AD and PD, fly models have been generated by mis-expression of mutant forms of human polyQ genes as well as loss- and gain-of-function of *Drosophila* homologues. In all of these cases, Gal4 specific mis-expression can cause pronounced neurodegenerative phenotypes in the eye or central nervous system that can be accompanied by aggregated formation and reduced life-span [for recent review, see 82,  208, 213]. Several studies established that toxicity is due to polyQ tracts [214, 215]. However, the protein context around the pathogenic expansion strongly modulates dominant toxicity, suggesting that the resulting aberrant protein conformation may trigger an amyloid-like cascade [216-218], and that protein-protein interactions are a major culprit of polyQ-related neurodegeneration. These protein-protein interactions occur even between polyQ-related disease proteins, at least for some of the proteins involved in SCA, thereby promoting neurodegeneration. For example, nuclear accumulation of ataxin-2 contributes to SCA1-related neurodegenerative phenotypes caused by ataxin-1[82Q], probably by direct interaction [219]. Ataxin-2 can also modify SCA3-induced neurodegeneration by accelerating the onset of nuclear inclusion formation associated with SCA3. Ataxin-2 activity depends on a conserved protein interaction domain, the PAM2 motif, which mediates binding of cytoplasmic poly(A)-binding protein which itself can influence SCA3-associated neurodegeneration [220].

Based on available fly models of polyQ-induced neurode-generation, numerous genetic and pharmacological modifier screens identified targets affecting protein folding and oligomerization [221-228], as well as RNA [229], microRNA [230] and RNA processing [228] as mediators of polyQ toxicity. These data suggest that altered protein conformation is only one of the causes underlying polyQ-induced neurodegeneration. However, the actual cause(s) of cell death are currently unknown. Apoptotic signalling [228, 231] has been involved, as well as the retinoblastoma pathway [232]; sumoylation and ubiquitination [233, 234], as well as PI3K/Akt [235] and TOR signalling affecting macroautophagy [236]. It is not clear whether these pathways are activated as a cause or consequence, but they do provide a target for therapeutic intervention. This is particularly evident for histone deacetylase (HDAC) inhibitors, which protect against polyQ toxicity [237, 238]. Interestingly, this effect can be modulated by simultaneous inhibition of two HDACs leading to enhanced neuroprotection [239], suggesting that HDACs may propagate/accelerate toxicity in *Drosophila *models of PolyQ-mediated neurodegeneration.

## FRAGILE X SYNDROME AND FRIEDREICH ATAXIA

Trinucleotide repeat expansion can also occur in non-coding regions, as is the case for FRX and FRDA. In both cases, single gene loci are affected and repeat expansion of >200 inversely correlates with age of onset. FRX occurs in 1/4000 males and 1/8000 females, leading to mental retardation and behavioural abnormalities due to expansion of unstable non-coding CGG repeats in the 5 prime untranslated region of *fragile X mental retardation 1* (*FMR1*). These excess repeats cause aberrant methylation of CpG islands and decreased histone acetylation in the 5 prime regulatory region of *FMR1*, leading to loss of *FMR1* and its encoded protein FMRP. FMRP is a selective RNA-binding protein that shuttles between nucleus and cytoplasm and controls local protein synthesis by suppressing mRNA translation (for review see [208]). This function of FMRP appears to be evolutionary conserved, as both mammalian FMRP and its *Drosophila* homologue *dfmr1* target the microtubule-associated protein 1B/*Drosophila* futsch [240]. Local regulation of protein synthesis is an essential function in synaptic terminals, and several studies in *Drosophila* identified related targets of *dfmr1*, including *rac1*, *pick pocket* [241, 242], *discs overgrown* and *polyA-binding protein* [243], all of which are implicated in synaptic function. Moreover, several loss- and gain-of-function studies demonstrated that *dfmr1* regulates synaptic structure [244] by associating physically and functionally with the microRNA pathway, thereby regulating the translation of synaptic mRNAs [for review see 245].

FRDA is an autosomal recessive disorder that occurs in 1/50,000 individuals characterised by gait ataxia due to progressive atrophy of dorsal columns, and spinocerebellar and corticospinal tracts. Unstable GAA repeats in the first intron of *FRDA* encoding frataxin inhibit transcriptional elongation, leading to decreased protein levels. Frataxin localises to the inner mitochondrial membrane and is involved in the regulation of iron levels and free radical protection. Similar functions have been identified for the only *Drosophila* homologue, *dfh* [246-248], suggesting that oxidative stress and impaired biosynthesis of Fe-S cluster containing proteins in the mitochondrial respiratory chain might be causally related to disease. However, as is the case for PD, it is still unclear whether and how oxidative stress can cause cell death, or whether it is merely a potentiator of mitochondrial dysfunction, ultimately leading to neurodegeneration. The available *Drosophila* model of FRDA will certainly allow rigorous testing of several hypotheses, including the possibility that impaired oxidative phosphorylation due to frataxin deficiency might be the major culprit underlying FRDA pathogenesis.

## MOTOR NEURON DISEASES

MND is a common denominator for several etiologically heterogeneous diseases affecting upper motor neurons located principally in the primary motor cortex and/or lower motor neurons located in the anterior horn of the spinal cord and the brainstem. MNDs include amyotrophic lateral sclerosis (ALS), some forms of FTD and fronto-temporal lobar degeneration (FTLD), as well as spinal muscular atrophy (SMA), hereditary spastic paraplegia (HSP), and others [249-254]. The most prevalent cases are SMA (1/6,000-10,000), HSP (3-10/100,000), and ALS (4-6/100,000). MNDs vary in severity and can affect all ages from infancy (SMA) to late onset (FTD and FTLD). However, in every case, the degeneration of motor neurons causes neuronal dysfunction and muscle wasting, leading to various locomotor disabilities, such as inability to use arms and/or legs, to speak or to swallow, and ultimately can cause the death of the patient when breathing muscles become involved.

**Fig. (4):**
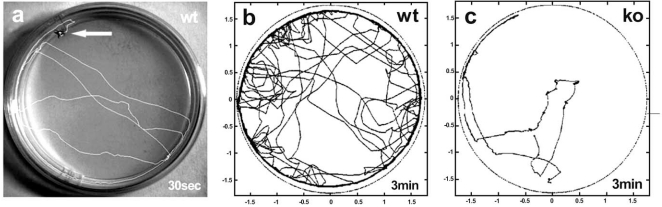
**Experimental study of *Drosophila* locomotor behaviour**. (**a**) An adult wild-type fly (*wt*, arrow) is kept in an arena which can be a converted petri dish. The fly’s activity and movement is recorded with a high-speed video camera and a computer programme tracks the resulting trajectory during a given time-window (30sec). (**b**) 3min movement trajectory of a *wt* fly; (**c**) 3min trajectory of a mutant fly (*ko*) revealing motor deficits. Video-assisted movement tracking records locomotor behaviour and in turn allows the quantification of parameters that can be used to describe it, including walking activity, velocity, and distance travelled. By applying this method to *Drosophila* models of neurodegeneration, it is possible to mimic adult-onset neurodegenerative movement disorders including Parkinson’s disease, trinucleotide repeat expansion diseases, and motor neuron diseases, and to monitor their effect on neural circuits and behaviour, which in turn allows genetic dissection of the underlying pathogenic mechanism(s).

To date, there is no effective therapy or cure for MND. As is the case for AD and PD, the majority of MND cases are sporadic and the causes are unknown. However, the identification of protein aggregates (fragmented, phosphorylated, ubiquitinated) in intracellular inclusions as well as the analyses of heritable, familial cases led to the identification of genes involved in MND pathogenesis (Table **4**). In some cases motor neuron death is caused by mutations in a single gene, such as *survival of motor neurons (SMN)* causing SMA [255]. For the majority of these disease-related genes, *Drosophila* homologues are present (Table **4**) and movement disorders can be easily monitored in the fly (Fig. **4**). Thus, loss- and gain-of-function analyses or mis-expression of the human disease gene have been used to establish fly models of MND.

## AMYOTROPHIC LATERAL SCLEROSIS

ALS is the most common adult-onset MND affecting upper and lower motor neurons, with an age of onset between 40 and 60 years. The majority of ALS cases are sporadic with currently unknown causes whereas 5-10% are familial cases (FALS), for which several disease-related genes have been identified [253, 254]. Most prominent among them are mutations of the Cu/Zn superoxide dismutase SOD1 which account for 15-20% of autosomal dominant FALS cases and 1-2% of all ALS cases. Although implicated in anti-oxidative activity, the precise role of SOD1 dysfunction in ALS is incompletely understood [256]. *Drosophila* models of SOD1 include mis-expression of human FALS forms as well as loss-and gain-of-function of the *Drosophila* SOD1 homologue [257-261].

Surprisingly and in contrast to the human condition, initial studies in *Drosophila* showed that expression of either *Drosophila* or human SOD1 results in increased lifespan without affecting locomotion or motor neuron survival [258-260], whereas loss of *Drosophila* SOD1 decreases lifespan [260] and causes necrotic cell death in the fly eye [257]. Yet, a recent study suggests that motor neuron-specific expression of wild-type or disease-linked (A4V, G85R) mutants of human SOD1 do not affect lifespan but induce progressive climbing defects that are accompanied by impaired neural circuit physiology and a stress response in surrounding glial cells [261]. The observed phenotypes occurred without loss of motor neurons but were accompanied with an age-related accumulation of mutant human SOD1-specific aggregates. These data, together with earlier results [257] raise the interesting possibility that SOD1 mutations leading to soluble oligomers rather than aggregate formation confer cellular toxicity – as seen for Abeta in AD and for ASYN in PD.

In addition to SOD1, TDP-43 was recently identified as a major player in the pathogenesis of ALS and probably other neurodegenerative diseases. TDP-43 encodes a primarily nuclear protein with so far unknown function(s) in the nervous system [262]. Phosphorylated and ubiquitinated C-terminal fragments of TDP-43 are found as cytoplasmic inclusions not only in ALS and FTLD [15, 16], but also in AD and PD [263]. Significantly, TDP-43 mutations affecting the C-terminal part of the protein have been found in both sporadic ALS and FALS (for review, see [264]), suggesting that cytoplasmic accumulation and/or nuclear loss-of-function of TDP-43 is causally related to MND formation. *Drosophila* encodes two homologues of TDP-43 (Table **4**) and three recent publications provide initial insights into the function of one of these homologues [265-267]. Two of these studies show that hypomorhpic TBPH alleles lead to reduced lifespan and locomotor defects [265,266]. As potential causes, changes in the number of synapses at the larval neuromuscular junction (NMJ) [265] and defective dendritic pruning of larval sensory neurons [266] are reported. A third study describes a TBPH deletion which causes larval lethality accompanied by reduced HDAC6 levels, a molecular phenotype also seen in HEK293E cells depleted for TDP-43 [267]. In addition, a recent study over-expressed human forms of wild-type and mutant TDP-43 that where C-terminally tagged with red fluorescent protein (RFP). Mis-expression of these constructs led to degenerative phenotypes in the eye and central brain [268], but it remains to be seen whether these phenotypes are attributable to gain of TDP-43 function or rather to RFP. Moreover, insights into tissue-specific gene expression or potential pathogenic mechanisms underlying TDP-43-related adult-onset, age-related MND are still lacking.

Fly models of ALS also exist for vesicle-associated membrane protein B (VAPB) which is involved in rare cases of late-onset ALS. The VAPB P56S mutation is nevertheless of interest because it was found in ALS and SMA cases, indicating a potential common pathogenic mechanism underlying both MNDs. Mis-expression of wild-type or mutant forms of human VAPB as well as loss-of-function of the corresponding *Drosophila* homologue VAP33-1 [269, 270] affect synapse formation and maintenance [269-271] and can lead to aggregate formation, motor neuron loss and locomotion defects. The underlying pathogenic mechanisms are only beginning to emerge. A recent study suggests that VAP33/VAPB leads to defective bone morphogenetic protein (BMP) signalling at the NMJ, eventually causing motor neurodegeneration [270], whereas another study demonstrated that VAPB is involved in Eph receptor signalling [272].

## SPINAL MUSCULAR ATROPHY

SMA is, after cystic fibrosis, the most common autosomal recessive disorder in humans and is characterised by loss of lower motor neurons and progressive muscular atrophy in the limbs and trunk, eventually leading to respiratory failure and death [250]. SMA is caused by recessive mutations of *SMN1* which, together with *SMN2* provides functional SMN required for motor neuron survival. Disease severity is inversely proportional to levels of SMN; however, the mechanistic details of a motor neuron requirement of SMN are incompletely understood [255]. *Drosophila* encodes a homologue of SMN and loss-of-function of dSMN results in recessive larval lethality and NMJ defects [273, 274] which appear to be due to a bidirectional function of dSMN in both muscles and neurons [275]. A subsequent genomic screen characterised enhancers and suppressors of dSMN and identified altered BMP signalling as a potential pathogenic pathway – a situation strikingly similar to the ALS-related VAP33/VAPB phenotype [270].

## HEREDITARY SPASTIC PARAPLEGIA

HSP describes a heterogeneous group of genetic disorders that are characterised by retrograde axonal degeneration of the corticospinal tracts and posterior columns in the spinal cord, leading to the loss of lower motor neurons and subsequent progressive spasticity and weakness of the lower limbs [276]. Several genetic loci have been identified that are causally related to HSP formation (for a recent update see [254]). The majority of cases are caused by mutations in Spastin which account for 40% of autosomal dominant HSP [277]. A *Drosophila* homologue has been identified [278] and several studies addressing dSpastin loss-of-function phenotypes as well as over-expression of human mutant forms established a role in microtubule organisation/stability, synapse morphology and neurotransmitter release at the NMJ [279-283]. Insights into the underlying pathogenic pathway(s) are coming from studies addressing the function of two other HSP-related genes, *Atlastin-1 (Atl-1)* and *non-imprinted in Prader-Willi/Angelman syndrome 1* (*NIPA1*).

*Drosophila* Atl localizes to endoplasmic reticulum membranes and mediates membrane tethering and fusion [284]; it is also expressed in muscles and functions with Spastin in microtubule organisation/stability [285]. Genetic analyses revealed that *Drosophila* Atl is required for normal growth of muscles and synapses at the NMJ as loss-of-function mutations lead to reduced muscle size and increased numbers of synaptic boutons. Defects are accompanied by altered expression levels of synaptic proteins Dlg and alpha-spectrin and can be rescued by muscle but not neuron-specific Atl expression [285], suggesting that Atl and Spastin regulate synapse morphology at the NMJ *via *microtubule organisation/stability. Loss-and gain-of-function mutations of Spichthyin (Spict), the *Drosophila* homologue of human NIPA1, revealed that Spict regulates microtubule maintenance and morphology of presynaptic NMJs by way of BMP signalling, where it interacts with BMP receptors and promotes their internalization from the plasma membrane [286]. A recent follow-up study showed that mammalian NIPA1 also interacts with BMP type II receptor and similarly inhibits BMP signalling by regulating BMP receptor traffic [287]. Moreover, this study also provides evidence that *spastin* and another HSP-related gene *spartin* are also inhibitors of BMP signalling [287], indicating an evolutionary conserved role of Spast, Spict/NIPA1 and other HSP-related proteins in the regulation of BMP-mediated synapse morphology at the NMJ.

It is becoming apparent from these *Drosophila* studies into human MND that mutations of fly homologues of VAPB [270], SMN [275], NIPA1 and Spastin [286, 287] all seem to activate the same pathogenic pathway, suggesting that de-regulated BMP type II receptor signalling may unify ALS, SMA and HSP. This would make sense because all three MNDs affect lower motor neurons and BMP activity has been shown to play a significant role in the development and function of the NMJ [for review see 288, 289]. It will be interesting to see whether other MND-related genes also impinge on BMP signalling and whether its alteration is a cause or consequence of progressive motor neuron degeneration.

## CONCLUDING REMARKS AND OUTLOOK

The above mentioned studies exemplify *Drosophila* research over the past two decades in the study of human neurodegeneration which led to the establishment of reliable models for Alzheimer’s, Parkinson’s, and motor neuron diseases, as well as models for Trinucleotide repeat expansion diseases. These studies made valuable contributions to our understanding of the molecular, genetic, and cellular aspects of neurodegeneration from genes to brain and behaviour. However, three major aspects still remain elusive and further efforts are required to address them.

## WHAT CAUSES CELLULAR DEGENERATION?

The majority of neurodegenerative diseases are associated with the accumulation of misfolded proteins into aggregates that contain fibrillar structures. Although still debated, the prevalent hypothesis is that aggregates represent a cellular protection mechanism against toxic aggregation intermediates, whereas soluble oligomers and pre-fibrillar species most probably are the major cause of toxicity [115, 290, 291]. Several studies in *Drosophila* support this hypothesis [192, 193, 216, 257, 261]. However, the exact nature of the toxic species and how they exert their pathogenic function is still unclear. Thus, potential toxic species need to be tested in available *Drosophila* models (see [192]), followed by unbiased genome-wide modifier screens to identify molecular pathways executing cellular toxicity. In addition, there is mounting evidence suggesting that several of these toxic species, including forms of Abeta, Tau, ASYN, and HTT may harbour prion-like features that can trigger cell-to-cell transmission [292], thereby acting both as seeds of disease formation* and* propagators of disease progression. These features are compatible with *permissive templating* [293] and provide an explanation for the spread of disease from initially local foci to major regions of the brain, as seen for example in AD and PD [89,90]. Significantly, *permissive templating *represents an experimentally testable hypothesis in the search for additional prionogenic proteins [294] that may propagate disease progression. It remains to be tested whether cell-to-cell transmission of proteinopathies can be modelled in *Drosophila*, but a combination of bioinformatic and genetic modifier screens may provide powerful tools for experimental analyses.

## WHAT MEDIATES AGE-RELATED PATHOGENESIS?

The vast majority of patients suffering from neurodegenerative diseases represent sporadic cases. The post-mortem brain of such patients is usually characterised by inclusions composed of misfolded but non-mutated proteins where the cause of pathogenesis remains elusive. Several risk factors have been implicated in disease formation and progression, including environmental and genetic risk factor(s), but age remains the dominant, overarching one. Yet, almost nothing is known about age-related pathogenic mechansims and how they impact on neurodegeneration. Obvious suspects are genetic instability, decline in protein quality control and mitochondrial dysfunction [202, 203]. However, these age-related phenotypes may explain some but not all of the sporadic cases observed in neurodegenerative diseases. Thus, comparable to the multi-hit hypothesis on cancer [295], one has to postulate at least two or more initiating events that trigger disease formation in an age-related manner. This seems to be the case in *Drosophila*, where deregulation of two or more conserved signalling pathways is seen in models of genetically-induced proteinopathies. These include PI3K/Akt and TOR signalling in AD and PD, JNK signalling in AD and PD, histone acetylation in PD, MNDs and TREDs, as well as BMP signalling in MNDs. It is currently not clear whether de-regulated signalling represents a cause or consequence of disease, nor is it clear whether non-canonical pathway components are involved. However, the available genetic tools make it possible to investigate disease formation and progression in an *adult onset, age-related *manner in *Drosophila*, and to screen for unknown pathway components at a systematic genome-wide level (see [228]). This will allow genetic dissection of pathogenic pathways related to *age* as the main risk factor and to determine the causative roles of individual pathway components.

## WHAT PREVENTS DISEASE FORMATION?

An increased life expectancy in developed countries will steadily increase the number of individuals suffering from age-related neurodegenerative diseases. This represents an enormous socio-economic burden, and will intensify the demand to identify and develop new drugs and compounds that can be used for targeted treatment(s) in order to prevent or at least ameliorate disease symptoms. As part of these efforts, compounds need to be screened and tested in reliable models. *Drosophila* can make a significant contribution in this direction, and several successful examples have already shown that pharmacological intervention can ameliorate the disease, as demonstrated for example by the first description of geldanamycin and chaperone treatment of ASYN toxicity [68, 153]. Examples like this can directly guide clinical research and the development of novel therapeutic strategies for the treatment of these debilitating diseases. Considering previous work over the last two decades, it is reasonable to assume that *Drosophila* research will continue to make significant contributions in the study of human neurodegeneration.

## Figures and Tables

**Table 1:** Alzheimer’s Disease-Associated Genes

Gene/Protein	Inheritance	Fly Homolog	Protein Function
APP	AR	Appl/CG7727	Pre-synaptic protein
PSN-1/2	AR	dPs/CG18803	Gamma-secretase activity
Tau	unclear	tau/CG31057	Microtubule stabilization
APOe4	unclear	None	Lipid/cholesterol metabolism

APP = amyloid precursor protein; Appl = amyloid precursor protein-like; PSN-1/2 = presenilin-1/2; dPS = *Drosophila presenilin*; APOe4 = apolipoprotein e4; AR = autosomal recessive.

**Table 2:** Parkinson’s Disease-Associated Genes

Gene/Protein	Inheritance	Fly Homolog	Protein Function
Alpha-synuclein	AD	None	Pre-synaptic protein
Parkin	AR	parkin/CG10523	E3 ubiquitin ligase
UCH-L1	unclear	Uch/CG4265	E3 ubiquitin hydrolase/ligase
PINK1	AR	Pink1/CG4523	Mitochondrial kinase
DJ-1	AR	DJ-1a/CG6646 DJ-1b/CG1349	Redox sensor/Chaperone
LRRK2	AD	lrrk2/CG5483	Kinase/GTPase
HtrA2	AD	HtrA2/CG8486	Mitochondrial pro-apoptotic protease
GBA	unclear	CG33090	Lysosomal enzyme
POLG	unclear	tamas/CG8987	Mitochondrial DNA polymerase
Tau	unclear	tau/CG31057	Microtubule stabilisation

UCH-L1 = ubiquitin carboxyl-terminal esterase L1; PINK1 = PTEN induced putative kinase 1; LRRK2 = leucine-rich repeat kinase 2; HtrA2 = high temperature requirement protein A2; glucocerebrosidase = GBA; POLG = polymerase gamma; AD = autosomal dominant; AR = autosomal recessive.

**Table 3:** Trinucleotide Repeat Expansion Disease-Associated Genes

TRED	Gene/Protein	Inheritance	Fly Homolog	Protein Function
HD	HTT	AD	htt/CG9995	Microtubule binding, transport
SCA	ATXN1/2/3/7	AD	Atx-1/CG4547 Atx2/CG5166	unknown
SCA17	TBP	AD	Tbp/CG9874	Transcriptional regulation
SBMA	AR	AD	None	Nuclear receptor
DRPLA	ATN1	AD	Gug/CG6964	Transcriptional regulation
FRX	FMR1/2	X and AD	dFMR1/CG6203	RNA regulation
FRDA	FXN	AR	fh/CG8971	Mitochondrial protein

TRED = trinucleotide repeat expansion disease; HD = Huntington’s disease; SBMA = spinal bulbar muscular atrophy; SCA = spinocerebellar ataxias; DRPLA = dentatorubropallidoluysian atrophy; FRX = fragile X syndrome; FRDA = Friedreich’s ataxia; HTT = Huntingtin; ATXN-1/2/3/7 = ataxin-1/2/3/7; AR = androgen receptor; TBP = TATA box binding protein; CACNA1A = calcium channel, voltage-dependent, P/Q type, alpha 1A subunit; ATN1 = atrophin -1; Gug = Grunge; FMR1/2 = fragile X mental retardation 1; FXN = frataxin; fh = frataxin homolog; AD = autosomal dominant; X = X-linked chromosomal segregation; AR = autosomal recessive.

**Table 4:** Motor Neuron Disease-Associated Genes

MND	Gene/Protein	Inheritance	Fly Homolog	Protein Function
ALS	SOD1	AD/(AR)	Sod/CG11793	Superoxide dismutase
ALS	Alsin	AR	CG7158	unknown
ALS	SETX	AD	None	DNA/RNA helicase
ALS	FUS/TLS	AD/(AR)	caz/CG3606	Transcription/RNA processing
ALS	VAPB	AD	Vap-33-1/CG5014	Cargo transport
ALS	TDP-43	AD	TBPH/CG10327 CG7804	Transcription/RNA processing
ALS	CHMP2B	unclear	CG4618	Endosomal sorting/transport
SMA	SMN-1/2	AR	Smn/CG16725	Transcription/RNA processing
HSP	SPAST	AD	dSpast/CG5977	Microtubule organisation
HSP	NIPA1	AD	spict/CG12292	Synaptic growth/BMP signalling
HSP	ATL-1	AD	atl/CG6668	Membrane fusion/ER

MND = motor neuron disease; ALS = amyotrophic lateral sclerosis; SMA = spinal muscular atrophy; HSP = hereditary spastic paraplegia; SOD1 = Cu/Zn superoxide dismutase 1; SETX = senataxin; FUS/TLS = fused in sarcoma/translocated in liposarcoma; caz = cabeza; VAPB = vesicle-associated membrane protein B; Vap-33-1 = vesicle-associated membrane protein 33-1; TDP-43 = transactive response DNA-binding protein 43; TBPH = transactive response DNA-binding protein homologue; CHMP2B = charged multivesicular body protein 2B; SMN-1/2 = survival of motor neuron protein 1/2; SPAST = spastin; NIPA1 = non-imprinted in Prader-Willi/Angelman syndrome 1; spict = spichthyin; ATL-1 = atlastin-1; ER = endoplasmatic reticulum; AD = autosomal dominant; AR = autosomal recessive.

## ABBREVIATIONS

AD: = Alzheimer’s disease
ALS: = Amyotrophic lateral sclerosis
APOe4: = Apolipoprotein e4
APP: = Amyloid precursor protein
Appl: = Amyloid precursor protein-like
AR: = Androgen receptor
ASYN: = Alpha-synuclein
ATL-1: = Atlastin-1
ATP: = Adenosin tri-phosphate
ATN1: = Atrophin -1
ATXN-1/2/3/7: = Ataxin-1/2/3/7
BACE: = Beta-site APP-cleaving enzyme
BMP: = Bone morphogenetic protein
CACNA1A: = Calcium channel, voltage-dependent, P/Q type, alpha 1A subunit
Caz: = Cabeza
CHMP2B: = Charged multivesicular body protein 2B
CNS: = Central Nervous System
DA: = Dopamine
DNA: = Desoxy ribonucleic acid
DRPLA: = Dentatorubropallidoluysian Atrophy
ER: = Endoplasmatic reticulum
FAD: = Familial AD
FALS: = Familial ALS
Fh: = Frataxin homolog
FMR1/2: = Fragile X mental retardation 1
FRDA: = Friedreich’s ataxia
FRX: = Fragile X syndrome
FTD: = Fronto-temporal dementia
FTLD: = Fronto-temporal lobar degeneration
FUS/TLS: = Fused in sarcoma/translocated in liposarcoma
FXN: = Frataxin
GABA: = Gamma-aminobutyric acid
GFP: = Green Fluorescent Protein
Gug: = Grunge
HD: = Huntington’s disease
HDAC: = Histone deacetylase
HSP: = Hereditary spastic paraplegia
Hsp70: = Heat shock protein 70
HtrA2: = High temperature requirement protein A2
HTT: = Huntingtin
JNK: = c-Jun N-terminal kinase
LRRK2: = Leucine-rich repeat kinase 2
MND: = Motor neuron diseases
MPTP: = 1-Methyl-4-phenyl-1,2,3,6-tetrahydropyridine
mtDNA: = Mitochondrial DNA
NIPA1: = Non-imprinted in Prader-Willi/Angelman syndrome 1
NMJ: = Neuromuscular junction
PD: = Parkinson’s disease
PI3K: = Phosphatidylinositol 3-kinase
PINK1: = Phosphatase and tensin homologue (PTEN)- induced kinase 1
POLG: = Polymerase gamma
PolyQ: = Polyglutamine
PSN-1/2: = Presenilin-1/2
RFP: = Red Fluorescent Protein
RNA: = Ribonucleic Acid
SBMA: = Spinal Bulbar Muscular Atrophy
SCA: = Spinocerebellar ataxias
SETX: = Senataxin
SMA: = Spinal muscular atrophy
SMN-1/2: = Survival of motor neuron protein 1/2
SOD1: = Cu/Zn superoxide dismutase 1
SPAST: = Spastin
Spict: = Spichthyin
TBP: = TATA box binding protein
TBPH: = Transactive response DNA-binding protein homologue
TDP-43: = TAR DNA binding protein 43
TOR: = Target of rapamycin
TRED: = Trinucleotide repeat expansion diseases
UAS: = Upstream activating sequence
Vap-33-1: = Vesicle-associated membrane protein 33-1
VAPB: = Vesicle-associated membrane protein B
